# Editorial Correspondence

**Published:** 1877-05

**Authors:** E. F. Starr

**Affiliations:** Nacoochee, Georgia


### EDITORIAL CORRESPONDENCE.

NACOOCHEE, GEORGIA.

*Dr. J. G. Westmoreland:*

DEAR SIR—Allow me in this note to commend your article, and views, as expressed in the March number of this JOURNAL, on the subject of meningitis, and to say that, if my experience is worth anything, and if I have been able to draw correct conclusions from my observations in intercranial inflammations, these all go to confirm the position which you seem to occupy on this important subject.

I am one of the minority with which you allign yourself, not from favoritism nor preconceptions, but from both experience and theory. Then, of the "distinguished medical authority" on the other side let me ask, are they distinguished for their success in the treatment of this disease? If not, how is the tree to be known? Where is the fruit? I was talking with one of these distinguished gentlemen, some time ago, on this subject, and was advocating the use of the lancet. "Do you bleed," he asked, "for meningitis?" I answered emphatically, yes, and cited a case which I had treated not long before—a very distinct and well marked case of the cerebro-spinal disease, in a boy five years old—in which I practiced general bleeding three times in the course of a day and a half, which, with auxiliary treatment, resulted in the relief of the alarming symptoms and the recovery of the patient. "Well, now," said he, "to tell you the truth, the only case of it I ever cured was a case that I bled." On the next visit to this case, after the first bleeding, and administration of morphine and calomel, it was gratifying to observe the improvement in the symptoms, and especially in the pulse, which, from small and frequent, had changed to rounder, fuller, and much less frequent. In short, the pressure upon, or the oppression of, the nervous centres having been considerably relieved, the case had been changed from an apparently asthenic, into one of an opposite character. But this was not enough; the then sthenic condition had to be watched and treated, and the restraining force had to be continued until the inflammatory condition yielded. I have not kept a record of my cases, but of about ten cases that I can now call to mind, of cerebral and cerebro-spinal meningitis treated with venesection as one of the remedies, seven have recovered and three died. Of these three, one died of relapse brought on by over exertion, after having been fairly and hopefully convalescent, and the other two were not bled soon enough, or in the most propitious time; while of five cases that I have known, not treated with venesection, all have died. I will not say that the general abstraction of blood is absolutely necessary, or called for, in all cases of meningitis, but in such cases as have fallen under my observation, I think it was generally a *sine qua non.*

Since your article was published, another has appeared from Prof. Banks, (see this JOURNAL for April,) in which he says: "I am forced to regard it as general disease, dependent upon some unknown epidemic influence—a malignant epidemic fever," etc. My observation has led me to believe that it is most frequently caused either by exposure to inclement weather, or by the peculiar epidemic influence which results in that disease of protean features known as *influenza;* and that meningitis is one of its ways of manifesting itself, just as bronchitis, tonsilitis, pleuritis, pneumonitis, peritonitis, cellulitis, erysipelas, neuralgia, spinal rheumatism, etc., are some of its ways of manifesting itself, and that it (meningitis attending influenza) is a "general disease," and a "malignant epidemic fever," to this extent, and to this extent only. But I will venture the prediction that, to such as consider it to be, and treat it as, a malignant epidemic fever, ignoring the importance of the local symptoms and condition, it will continue to prove an *opprobrium medicorum.* Does this form of disease occur without head symptoms of a grave character, as an early and prominent feature of the attack? May not an overwhelming irritative engorgement, as a first stage of inflammation, depress the vital functions, and even destroy life, before time has been given for the plainer lesions of developed inflammation? I think it may, yet this should not prevent it being properly called a local inflammation. Some seem to strive to lose sight of the local symptoms and condition, while contemplating the supposed malignity of a general disease, for Dr. Banks says: "The disease being a specific malignant fever, the usual lesions found after death should be regarded as secondary, and not as a primary and local inflammation;" and again, "the rapid fatal results, often before any structural lesions can be found, demonstrate the existence of an active malignant case," etc. "Local lesions" occur, but they are "commonly developed as the disease progresses," scarcely by inflammation, but by malignancy perhaps. My opinion is, that inflammation oftener produces lesions and milignancy, than malignancy produces inflammation and lesions. Be this as it may — for the good of humanity, for the credit and honor of the profession, and for the advancement of science, this question should be settled. Let the discussion continue to that end. In conclusion, it appears to me we have too many contagions, too much septicism, too many bacteria, too much mysterious malignancy, in these days, for the good of medicine. But

"Truth crushed to earth will rise again."

Yours, truly, E. F. STARR, M. D.

